# OSCE rater cognition – an international multi-centre qualitative study

**DOI:** 10.1186/s12909-021-03077-w

**Published:** 2022-01-03

**Authors:** Sarah Hyde, Christine Fessey, Katharine Boursicot, Rhoda MacKenzie, Deirdre McGrath

**Affiliations:** 1 School of Medicine at the University of Limerick, Health Research Institute, Limerick, Ireland; 2 St George’s Medical School, University of London, London, UK; 3 Health Professional Assessment Consultancy, Singapore, Singapore; 4 University of Aberdeen, Aberdeen, UK

**Keywords:** Clinical education, Competence assessment, Rater-based assessment, Rater judgements, Rating process

## Abstract

**Introduction:**

This study aimed to explore the decision-making processes of raters during objective structured clinical examinations (OSCEs), in particular to explore the tacit assumptions and beliefs of raters as well as rater idiosyncrasies.

**Methods:**

Thinking aloud protocol interviews were used to gather data on the thoughts of examiners during their decision-making, while watching trigger OSCE videos and rating candidates. A purposeful recruiting strategy was taken, with a view to interviewing both examiners with many years of experience (greater than six years) and those with less experience examining at final medical examination level.

**Results:**

Thirty-one interviews were conducted in three centres in three different countries. Three themes were identified during data analysis, entitled ‘OSCEs are inauthentic’, ‘looking for glimpses of truth’ and ‘evolution with experience’.

**Conclusion:**

Raters perceive that the shortcomings of OSCEs can have unwanted effects on student behaviour. Some examiners, more likely the more experienced group, may deviate from an organisations directions due to perceived shortcomings of the assessment. No method of assessment is without flaw, and it is important to be aware of the limitations and shortcomings of assessment methods on student performance and examiner perception. Further study of assessor and student perception of OSCE performance would be helpful.

## Introduction

Objective structured clinical examinations (OSCEs) rely on assessment judgements. It is known that factors other than candidate performance at OSCE stations can influence grading decisions, including factors related to raters [1]. Fairness and defensibility of examinations demands that ‘sources of error’ are addressed, so that examinations might become more reliable. This is particularly important for high stakes examinations. Various strategies have therefore been employed to mitigate for the issue of low interrater reliability, including training of examiners and the use of very structured checklists to minimise the effect of bias. These approaches have met with limited success [2, 3].

In the case of examiner training, this has generally been an attempt to educate the examiners as to an organisation’s standards and protocols [4]. This aims to reduce examiners’ dependence on personal experience and promote more accurate scoring. There is limited evidence as to whether training of examiners is effective in improving scoring reliability or accuracy [5, 6] A randomised controlled trial evaluating a rater training workshop showed that there was no benefit to training raters except in terms of rater confidence [2]. The use of very detailed checklists has also been used in an effort to reduce the ‘subjectivity’ of an examiners rating [3]. However extensive checklists can cause cognitive overload and cause an examiner to rely on memory, or to simplify a task by prioritising potentially idiosyncratic elements of the performance [7–9].

Rather than trying to change how examiners examine, it has been argued that a better approach might be to understand how and why examiners come to their conclusions. Rating, once believed to be a partially passive activity, is now understood to be an active cognitive process [10] requiring considerable mental workload [11]. This realisation has led to increased interest in rater cognition. It is known that raters’ scores vary considerably, but it is not fully understood why. Gingerich et al. described three prevalent perspectives on the origins of variability in assessment judgements; the assessor as trainable, the assessor as fallible and the assessor as meaningfully idiosyncratic [12]. Yeates et al. [7] looked to elucidate the mechanisms contributing to variability in scoring between raters by considering the interplay of social and cognitive factors. Three main mechanisms contributing to variability were described; differential salience (valuing/prioritising different aspects of a performance), criterion uncertainty and, information integration. A more recent study demonstrated that such assessments depend on which aspects of a performance an examiner prioritises, physician examiners tend to prioritise rapport-building or medical expertise skills [10]. Despite recent progress, there is still uncertainty over how to deal with rater inconsistencies during OSCEs.

It is possible that examiners develop their own tacit constructs which may affect their perceptions of a trainee’s performance [13]. This study aimed to explore the implicit, preconceived and the tacit responses of OSCE examiners in order to shine further light on how raters make their decisions. These tacit constructs may be acquired through a process of socialisation [13] and may be affected by local norms. For this reason, a multi-centre international study was designed. OSCEs rely on rater judgements and final year OSCEs were selected as the context for this study. This is a high stakes examination at an important threshold. Exploration of examiners’ preexisting tacit beliefs may help better understand how rater differences occur.

The aims of this study were:

1. To explore the tacit assumptions and beliefs of raters.
2. To explore rater idiosyncrasies.
3. To explore the decision-making process of examiners during OSCEs, how raters form impressions and make judgements about student performances.

## Methods

### Research strategy and research method

Starting from a viewpoint that reality is socially constructed and that a rater’s perception of student performance is informed by many interrelated elements, this study adopted a constructivist perspective to investigate the complex phenomena of rater cognition during an OSCE [14]. Qualitative methods aimed to provide an understanding of examiners’ decision-making during a rating exercise. Thinking-aloud interviews were used to gather data on the thoughts of examiners as they watched trigger videos of candidates. The process was iterative, involved purposeful sampling and a constant comparison approach to data analysis [15].

### Research context

Three investigators conducted between 9 and 11 individual interviews in three different centres. The centres were in England, Scotland and Ireland. Examiners were consultant-level doctors with experience examining at final year medical examinations. Semi-structured interviews with trigger videos were used to gather data. These were training videos produced by the University of Aberdeen Medical School. A purposeful recruiting strategy was taken with a view to interviewing both examiners with many years of experience, and those with less experience examining at final medical examination level. The interviews were conducted by a different interviewer in each centre, however the interviewers were trained and a number of interviews were supervised by one of the principal investigators. Informed consent was attained and all interviews were audio-recorded. Interviews were then transcribed. Participants received no financial compensation for their participation. The study was granted ethical approval by Ethics Committees at each of the three centres.

### Data collection

Each examiner was interviewed individually. Interviews lasted approximately 45 min and were audiotaped and transcribed. Examiners chose two simulated OSCE trigger videos to mark, from a choice of five history taking stations, and from a choice of four examination stations. Participants selected one history station and one physical examination station. The trigger videos were five minutes in length. All of the trigger videos showed competent candidates. Participants were allowed to choose from a small selection of trigger videos to ensure that they were watching an OSCE that they would feel competent to examine. Prior to watching the trigger videos, a short warm up video was shown, and participants were encouraged to ‘think aloud’ or describe out loud what they were thinking as they watched the student performance.

Examiners observed each trigger video twice, first in one uninterrupted run-through sequence. This aimed to capture the first impressions of the examiner in order to elicit features of the examiner’s thinking under usual OSCE conditions. Examiners then participated in a second (interrupted) viewing of the material and were invited to pause the video and expand on their impressions of the candidate’s performance. This afforded the examiner an opportunity to identify key elements of candidate performance and to say more about it. The interviewer used conversational interview techniques to elicit responses and also more detailed and deliberative responses during the second showing of the video. Examiners were asked to give a global score to the candidate in each trigger video. Questions aimed to expand on what the examiners had already identified - typically issues concerned with candidate progression, candidate knowledge and level of skill as shown explicitly, examiner decision certainty and examiners increased awareness of their inner transcript after completing the research exercise.

### Data analysis

Qualitative data analysis was conducted at the same time as data collection [15]. Data was analysed using a constant comparative method. Thematic analysis was used to analyse the talk aloud transcripts. The approach as outlined by Vaismoradi [16] and the phases of data analysis as per Braun and Clarke [17] were followed. Each transcript was reviewed by at least two investigators. Using an iterative approach preliminary codes were developed. NVivo was used for data analysis which allowed for an audit trail to be created. Reflexivity was achieved using a reflective diary, allowing identification of personal biases and tracking changes in perspectives by the analysts.

## Results

Thirty-one consultant level doctors participated in interviews, all of whom had recently been examiners at final medical examinations. Experienced examiners were classed as those examiners with over six years experience of examining at final medical exam level. Some examiners had over 20 years examining experience. A broad range of specialties was represented, with the majority being consultants (Attendings) in Medicine or Surgery and a smaller number from Psychiatry, Paediatrics, Academic Medicine, Obstetrics and Anaesthetics. The majority of experienced examiners had examined in varied institutions at undergraduate and postgraduate level. Demographic data is shown in Table 1.

**Table 1:** Demographic data

Centre	Gender	Level of experience
England	4 Male, 6 Female	7 experienced, 3 less experienced
Ireland	9 Male, 1 Female	9 experienced, 1 less experienced
Scotland	6 Male, 5 Female	6 experienced, 5 less experienced

Three main themes were identified: ‘OSCEs are inauthentic’, ‘Looking for Glimpses of truth’ and ‘Evolution with experience’. Representative quotes are identified by interview centre (01, 02 or 03) and the order in which participants were interviewed.

### Theme 1 OSCEs are inauthentic

While it is well known that OSCEs cannot be truly reflective of the real world or a clinical environment, it quickly became clear the extent to which raters consider OSCEs to be fundamentally flawed. Raters believe flaws are present in many aspects of the exam itself, the scenarios, the marking, and of most concern is the perceived negative influences these flaws have on student learning and behaviour. This firm belief has an influence on how raters think while watching and rating candidates during OSCEs.

Participants identify lack of authenticity as a major shortcoming of OSCEs. Raters outlined ways in which OSCEs lack authenticity, including scenarios not being realistic and stations not providing an accurate representation of clinical environments. Simulated patients (SPs) or real patients become overly compliant during examinations as they become accustomed to the technique. SPs responses to students can be variable throughout the day. There is a belief that OSCEs do not succeed in being relatable to real day-to-day work, many participants used the word ‘fake’ during the interviews.

*“You cannot simulate for an equivalent type situation in the clinical practice, there is no point in saying that an emergency down in the ED or on the floor or in the operating room is the same as an exam, there is no comparison.”* 02010

*“they’re so artificial, you know the things you’re asked to do in seven minutes are ludicrous in reality, you would not do that at all and it would be dangerous to do some of the things in seven minutes so you learn how to pass OSCEs as a person sitting them”* 01002

As well as the overall set-up lacking authenticity, many raters also cite issues with the ways that OSCEs are marked. Issues mentioned included technology, lengthy checklists, and uncertainty over the constructs being examined. Examiner fatigue and raters examining at stations that they have no experience of, or conversely are overly expert in, were other issues raised. There was a sense that not only the candidates, but also the examiners are going through motions by rote.

*“It is a tick-box exercise for the examiner, who isn’t really an examiner, but merely an observer”* 02001

Clinicians may feel that students can pass OSCEs without understanding what they are supposed to be doing; students can gain marks very easily on a checklist, even if they perform relatively poorly. OSCEs do not discriminate well between students. Marking systems do not allow proper rewarding of students who demonstrate excellence, and mediocre students are often unfairly rewarded because of the way OSCEs are marked. These issues provoked frustration from examiners. Of major concern is that weak students are able to pass OSCEs by going through steps correctly, even if examiners feel that the student does not understand what they are doing.

*“I suppose you could say in theory anyway the OSCE is very clear in what the marks are given for so maybe it’s very fair, but I sometimes think it’s not fair to the outstanding students, and maybe even the weaker students get too much marks for just getting the basics right”* 02004

*“It was so well rehearsed by the students, there was one student who came in and did it absolutely beautifully, and you could tell that this person just had a connection and understood what was going on and maybe had done an elective where they’d done this with patients a few times and the whole story flowed, whereas everybody else ticked the boxes. And they all did very well, because they ticked the boxes, and they explained the relevant points to the patients, but in one or two it was done brilliantly. But they didn’t excel for that because there was no box that said ‘did this person excel in how they did this?’”* 02001

The ease by which poorer students attain marks leads to a discrepancy between marks attained in an OSCE and how the student will later perform in a real life setting. Some reported that there was little correlation with how candidates will actually perform as a doctor. This is a fundamental area in which OSCEs are perceived to lack authenticity.

*“This is a person who you would not have confidence in as a doctor. He might know his stuff, he probably knows all his stuff but you would not have confidence as a doctor. But what I find difficult with OSCE is that there usually isn’t a place to record that, I mean if you go through the marking system, he would actually pass”* 02004

Student nerves were discussed by many raters. Not only are the stations and marking schemes felt to be problematic, but students are sometimes unable to give an accurate account of their skills due to nerves. Examiners know that stress can impact on student performance, but they don’t know whether the student will behave in a similar way under the stresses of a clinical environment, which were felt to be different to those of an OSCE. This is another source of discrepancy between performance seen in an OSCE and real life behaviour.

*“The stresses of an examination are much different and I think a lot of, I think more candidates under-perform on the day of the examination than over-perform on the day. I think that would be my humble opinion about it and especially in the clinical situation, not in a written examination. So for that reason I think that it’s good to give the benefit of the doubt”* 02010

Raters discussed how the shortcomings of OSCEs can have unwanted effects on student behaviour. One influence of OSCEs is how some students seem to tailor their learning towards passing the OSCE, rather than how they will need to perform in real life. Students are learning to pass examinations, rather than learning how to practice medicine.

*“even today I was teaching the first years and they didn’t want to know about how to put it together, they just wanted to know how the OSCE worked and how they got marks, and it’s very frustrating because were trying to teach them to be doctors, not OSCE-passers”* 03003

Students can be seen to put on a performance instead of properly engaging with patients.

*“Because this guy is very exam oriented, he’s just ticking all the boxes, he- I won’t say he lacks empathy but in this just video clip, probably he did demonstrate that, he’s not thinking about the patient. He’s thinking about what are the points he needs to cover. He didn’t ask his name. There was no kind of, interaction between the patient and the student, um, he just, he was just getting his findings ready to tell the examiner, so that he can score, he can score marks. He didn’t say bye to the patient.”* 03007

OSCEs can have an effect on student behaviour, not just during preparation for the OSCE or during the OSCE itself, but at other times. Raters discussed how students learn how to pass the exam rather than immerse themselves in learning the skills, and that the OSCE format encourages superficial learning. It was felt that students are following lists to tick off marks in their head, rather than approaching tasks in a systematic way. This represents an unforeseen and unwanted consequence of OSCEs. Raters noted that at times students were performing by rote, rather than responding to the issues of the patient in front of them.

*“I think that’s a risk of OSCEs actually, I think you can learn how to pass an OSCE as opposed to learning the content and how to actually work in reality”* 01002

*“He knows what he’s about but I’m concerned that he’s going by a list rather than thinking logically”* 03002

Overall the false nature of many aspects of OSCEs, and the effects of this on student learning and behaviour is prominent in rater’s minds while they are rating students. These obstacles lead examiners to search for glimpses of authenticity in an artificial environment.

### Theme 2 looking for glimpses of truth

The firmly held, but previously not fully examined or expressed, belief of raters that most aspects of an OSCE are ‘fake’ leads examiners to seek out glimpses of a student’s true ability. Raters search for authenticity and are on the look-out for students who are just going through the motions by rote. This can lead to examiners rewarding or penalising idiosyncratic elements of a student performance. Some look for evidence of experience via technical skill or familiarity with the clinical environment, others say the rapport a student builds with the patient is something that cannot be easily simulated. An important differentiator for some raters is if students are sticking rigidly to a mental checklist, indicating superficial understanding, or working through a station logically, indicating a deeper understanding. Some raters prioritise how safe a student would be as a newly qualified doctor. These factors can influence how stringent or lenient a rater is in their marking of a candidate.

In the falseness of the exam situation, a marker that a student has spent a lot of time on wards or with patients is sought by some examiners. Showing familiarity with how to approach patients, or evidence of having been present on wards, familiarity with how beds work etc., are noted as authentic signs of a students’ experience.

*“It’s all the little things that they do at the beginning that makes you think, ‘oh they know things, they’ve seen this, they’ve done this’. They know what they’re like, they automatically watch rounds, or automatically shake hands with the patient, introduce themselves, they just establish a kind of baseline of things, you know, professional things, that you’d expect people to do and you think, ‘oh this person gets it, off you go’!‘02003*

Some raters look for indications that a student is well practiced, this often relates to physical examination technique. This can help raters try to differentiate very good students from more average students. Raters value varying aspects of examination technique for example, watching an abdominal examination station, some have very specific comments about how they want students to ballot kidneys, or hand placement during abdominal examination, and others on the degree of exposure of the patient, or other aspects of the exam. It was discussed that students could achieve marks by carrying out the examination correctly but, whether a student is just following a checklist, or is engaged with the process and would actually pick up abnormal findings was important for many raters.

*“His percussion is good, which is quite discriminatory. Experienced students, as he clearly is, get a nice confident percussion technique with a nice, clear percussion note”* 01001

*“You can almost see them feel a liver edge really, and certainly when they’re percussing you should be able to hear it. But you can kind of tell whether they’ve got it or not really. Even if there’s no liver edge to feel really. you can tell that they would do, if it was there. It’s almost like a feeling thing, you kind of- it almost feels like a feeling thing yourself. So it’s not just a flat looking, it’s a 3D assessment somehow, that you’re making”* 01003

*“It would take a little more time than what he has done here, to, for the information to be transmitted from your fingers to your brain, that there was or wasn’t a thrill or a heave there. In this instance, you may say that that’s just a guy that’s just going through the motions, at this point because if there surely was something there and it’s subtle, it may take a few more seconds …to put your fingers between the ribs, and see which chamber which may be contributing to the, the heave and so forth, you know? Erm, but, he’s done the process”* 02001

Some raters use clues which indicate that a student has a lot of experience with patients. Raters look at how students interact with patients, with some prioritising rapport with patients over technical issues, and rewarding this in terms of marking.

*“I probably heavily, heavily weight people on how they interact with a patient. The way my mind works is that if they do that really well, I’m less likely to notice small issues with practical issues, whether they’ve missed a question or missed one tiny part of the examination but because overall I’d be thinking that this is actually a good candidate. Because they have actually got that first really critical part, they’ve got a rapport with the patient therefore they’re likely to get a good history where the patients relaxed they’re likely to do a good examination.”* 03001

*“Language is important isn’t it and people probably don’t realise the messages they’re giving with the language they use. More experienced students who’ve been around patients will use language differently, I think, so it’s subtle but it shows. They probably don’t realise they are doing it cos I suspect, I think the patients are the best feedback actually because they’ll respond differently to the way you speak to them”* 01002

Clues as to how the student will perform as a doctor upon qualification are sometimes noted by raters. Raters sometimes benchmark the student against recently qualified doctors and whether a student demonstrates that they would be safe, or unsafe, as a doctor. Markers that a student may be unsafe act like unofficial red flags.

*“I suppose in my head I’m thinking, is this is a guy who’s showing me what he can do on a manikin, or is this a guy who’s showing me what he can do in real life? And to me I was thinking, I would trust this guy to do the first assessment of a surgical patient with an acute abdomen erm, so Yeah that’s, at that level I would have thought it was”* 03001

*“I think, with any exam, I am fundamentally looking for the person that’s unsafe, and that might be because they’re too confident, they’re not looking after the patient, they’re doing things that make me think, ‘you’ve not been near patients’, or ‘you might make decisions that are over your ability’. These are the things that would really concern me. Obviously if he’d done every single thing wrong, then yes, I would probably say no, he fails, but I think he was a caring person who will learn, and that’s what you kinda want I think that’s what we’re trying to achieve from our, people leaving our school”* 03003

Some raters describe relying on their own gestalt during an OSCE, rather than sticking rigidly to a marking system. There is a sense that since the OSCE is so inauthentic, that a rater has to use their own criteria and instincts about student skills. Sometimes judgements are not based on what a rater actually observes, such as this rater making a prediction about a candidate’s likely behaviour were they not under the pressure of time.

*“I think my thought process with that student was that he was doing some things effectively but against the rush of time, so that was making him make some errors and if there had been an abnormality, I think he would have gone back to listen or to check”* 03003

These assumptions sometimes go beyond the scope of the exam and relate back to how students are taught. Raters sometimes explain a candidate’s shortcomings based on things that they have not seen. These examiners disregard the shortcomings they have identified in candidates because their own judgements hold more weight.

*“I don’t know what instructions he’s been given or how he’s been trained (…) I’m sure he would, because he’s very good I’m sure he would normally do the other things”* 01001

*“He used the prostate cancer as the first thing. I think that wasn’t his fault, that was whoever gave him the lectures on urology and he used benign prostatic hypertrophy and that’s also to blame whoever gave him the lectures on prostatic disease” 02010*

They can override parts of the marking system or exam if they feel that a good candidate is being unfairly disadvantaged by the constraints of the system, but may be less likely to do so for a student they do not perceive to be as genuine*.*

*“sometime you see a candidate who is fantastic in terms of their skill, OK, and the second candidate was very good in terms of his skill. But another candidate might be very poor with the patient. Or unpleasant to them, or something like that. And so they get marked down for that but there’d be some degree of compensation. And I’m not quite sure, that’s the bit, I’m not quite sure I’d want them as my doctor. I kinda try and resolve it, how would they be if there were my houseman, would they- that’s the kinda bottom line, am I giving them a score that reflects that? at the bottom line”* 01005

### Theme 3 evolution with experience

Raters tend to feel that OSCEs are a performance or a charade, and, in turn, they look for signs of what they consider authentically shows a student’s actual ability. These preexisting thoughts which examiners bring with them into an OSCE are likely to influence their marking. Raters also adapt to the nuances in the different OSCEs they are involved in, and bring this knowledge with them. Some differences became apparent between the experienced and less experienced raters. There is an unspoken fear in less experienced examiners that they may be letting an incompetent student progress. They wish to be seen to be doing everything correctly and stick more rigidly to the instructions. Conversely, there is a sense from more experienced examiners that they are aware of the failings of OSCEs, that they have developed their own idiosyncratic beliefs in what demonstrates authenticity and are comfortable with bending the instructions to some extent. Experienced examiners tend to feel that they are rating only one of a number of stations, added to that is the fact that they know that, even though these are final examinations, that students do not have to achieve perfection, that students will continue to develop and learn as newly qualified doctors.

The flaws of OSCEs may make them poorly discriminating, but some raters find ways to reveal what they believe is a students’ true ability. Some experienced raters develop their own strategies to allow differentiation between students. Less experienced examiners are less likely to deviate from instructions.

*“We all probably have our favourite things that we think want to be in there, and weight those more or less than perhaps we should do according to the checklist in front of us”* 01003 experienced examiner

*“So that’s a good incisive traditional medical student question, ‘what did the doctor give you last?’ Which I would always reward. Mightn’t be on the OSCE sheet”* 02002 experienced examiner

The experienced raters, through experience, accept the shortcomings of OSCEs and are more comfortable with their own idiosyncratic ways of looking for authenticity. Inexperienced examiners are still not sure and are torn, and more likely to strictly adhere to the instructions that they have been given.

*“this is maybe a more general comment- but when you have a list, as an examiner, with different things to pick off, and then they don’t do certain things, you know, how hard do you then come down on the student, for missing out signs? Or how much do you actually use that more intuitive, global impression that you have cos they maybe missed one or two, even three things? I do know people who, who would say, you know like with the previous candidate, ‘if you don’t ask about suicidal ideation in a depressed patient, then that’s a fail because that’s a question’. I personally would feel that is harsh if they perform well in other respects”* 03005 experienced examiner

*“When I’m examining I have to be fair, and I will only tick the boxes he has performed. I cannot tick the boxes, assuming that he will be fine”* 03007 less experienced

More experienced raters were also more likely to intervene and move a candidate on during an OSCE station if they felt that a candidate was wasting time. Less experienced raters described the frustration of watching students doing things during an OSCE that are not on the checklist and therefore not accruing marks.

*“Our OSCE training is that were impartial, but to bring out the best in students, which is actually what we want to do, you’re not impartial. It’s that you may facilitate them to be better, and that might mean that I would have moved that student along, and that’s not impartial. But I know that other examiners may not do that. But I kind of feel that my role is to see how good they are, and particularly in the final year, that I want to see what they’re like because I want to think, are they going to be good doctors?”* 03003 experienced examiner

*“You spend a disproportionate amount of time looking for them to say these things and I’m always aware in history that they can be doing good quality history taking, getting into more details, but they’ve already got the point. Even though it’s good, and they’re delving into ones you’ve mentioned, key word, or that’s the key question, you have your point”* 02006 less experienced

Examiners are aware that there are differences between how they and other examiners might rate candidates. Co-examining with first-time examiners was described as challenging as new examiners have a tendency to question every small detail of a candidate performance. Examiners were aware that their own methods of rating OSCE candidates had evolved over time.

*‘it was a nightmare working with him all day because every minute he was stopping me or one of the other examiners to ask a query, what should I do, should I fail them on this, or that or whatever, and you know it was a function of just, he wanted to be seen to do the everything right, he had probably this fear that the was going to release this unqualified person onto the community. So all reasonable, and he was doing all the right things, but same time, if hes after a few years of doing it, he’ll have a much more balances view of the whole thing’ 02005*

*‘the first year or two I would have been very very conscious of everything, all the boxes ticked, and being mechanical in how you did it. But now, I have a more overall view of, an even, I suppose, sit back and have a sense of the overall standard as the day goes by as well’ 02008*

Experienced examiners tend to feel that they are rating only one of a number of stations, added to that is the fact that they know that, even though these are final examinations, that students do not have to achieve perfection, that students will continue to develop and learn as newly qualified doctors.

*‘I expect them to learn more as an FY1 and FY2, that they’ve got the tools of the trade now but they actually fine tune them with real patients because not having responsibility and having volunteer patients here, there is an element of artificiality which limits their learning of nuances. They’ve got the tools, basic tools, and they can refine them later on’03002*

## Discussion

Holmboe et al. [18]states that in order to better understand rater idiosyncrasy, it makes sense to start by investigating what raters actually observe, experience and can comment on. Our study used think aloud methodology and qualitative analysis to explore how assessors rate and judge candidates during OSCEs. Of particular interest were the tacit components and aspects that examiners themselves were likely to have only partial awareness of. This methodology allowed assessors to describe their thoughts as they were presented with an OSCE station. Examiners were from three different academic institutions and geographic areas. Many of the more experienced examiners had experience not only at high stakes exit examinations in their own institution, but also examining in various institutions and at post-graduate examinations. Despite this wide geographic spread and exposure to different OSCEs with varying checklists, briefings, stations etc., the concern about the shortcomings and lack of authenticity of OSCEs was universal. There were no major discrepancies between comments made by examiners from the three different schools. The superficial and theatrical nature of OSCEs has been well described in the literature, and this study confirms that raters at the coalface of examining students perceive this as a major issue and one which filters their thoughts as they are rating [19].

This study showed that raters are concerned about the perceived adverse consequence of OSCEs, in particular the effect on student behaviour, in terms of relating poorly with patients and taking a superficial approach to learning. There is general agreement that medical students are not well prepared for working as doctors upon qualification [20]. It has been suggested that current methods of assessment can discourage students from becoming meaningfully involved in real patient care and promote a formulaic non patient centered approach [21]. It has been said that the behaviours encouraged in students in order to pass exams are at odds with good medical practice [19].

Despite, or perhaps because of, the assessment being flawed, examiners look for pointers of authenticity or flashes of reality during an OSCE. Raters prioritising idiosyncratic elements of student performance has previously been noted by Yeates et al. [7], who looked to elucidate the mechanisms contributing to variability in scoring between raters by considering the interplay of social and cognitive factors. This study shows that idiosyncrasy can arise from raters trying to mitigate for the falseness of an OSCE. Govarts et al. noted that schemas develop through experience that therefore significant variation between raters may arise. Kogan [22] demonstrated that faculty’s own clinical skills may be associated with their rating of trainees. It appears that raters often make and justify their decisions based on personal theories and constructs of performance which they have developed over time and experience and other complex factors. Cognitive factors as well as contextual factors influence rater judgements in workplace based assessment [23]. These theories and factors may diverge from those intended by the organisation. In our study raters were sometimes aware of the discrepancy between their rating behaviour and the directions of the organisation. Raters in this study, particularly experienced examiners, used their own methods to reward what they consider true demonstrations of good behaviour from students (taking into account student stage and other factors). These methods likely develop from experience of watching students in practice, seeing young doctors working and based on their personal clinical practice and experience. These experiences shape an examiner’s beliefs about what shows authenticity, and this is why beliefs differ between examiners and are a source of idiosyncrasy.

Although the study included a greater number of more experienced examiners than less experienced (experienced examiners being defined as having over 6 years examining at this level), there were some apparent differences between the groups which were consistent across the three centres. In this study less experienced OSCE raters were likely to judge more stringently than more experienced examiners. More experienced examiners tended to draw a broad overall impression of students at an earlier stage, being more willing to allow certain shortcomings from candidates if the overall picture was positive and tended to arrive at decisions with relative ease. Less experienced examiners tended to focus on small details of the examination and inserted caveats and reservations during the discussion, this group came to a decision but seemed less certain at times in doing so. It is possible that as experienced examiners, as well as knowing the standards of qualifying doctors, also have experience of OSCEs in various institutions at various levels and have become comfortable reconciling their opposing concerns over the falseness of OSCEs and a student’s true abilities. Experienced raters have an understanding of context (‘fake’ OSCE), look for flashes of authenticity, acknowledging the official directions of the institution but generally prioritising their own gestalt, in arriving at a final judgement.

This study differs from other studies in that fitness for practice was not the larger consideration. This was likely because the trigger videos showed relatively competent students performing OSCEs. This may be perceived by some as a weakness. However, it could be argued that most students pass OSCEs and examiners decide quite early if a candidate is going to pass. In this study the candidates were likely to be perceived as reaching the threshold for practice, therefore this consideration was attenuated, allowing a deeper exploration of the more nuanced decisions an examiner makes. A checklist or global score was not used during the study as it was important that assessors could select their own area of interest during a station.

Raters work within the constraints of the system, and sometime use idiosyncratic ways of dealing with the perceived shortcomings of it. This study shows that raters have their own tacit assumptions, and that the root of many of these relate back to their lack of faith in OSCEs. This study gave an insight into how raters approach OSCEs – from their opinion of the method itself and how they dealt with its perceived shortcomings. As these raters are aware that they are deviating from instruction, but feel they have good reason to do so, it is likely to be difficult to change this behaviour. It could be argued that increasing the frequency of direct observation of trainees [12] by different raters could mitigate for this idiosyncrasy and provide timely and useful feedback encouraging ‘assessment for learning’ rather than ‘assessment of learning’. No method of assessment is without flaw, but it is important to be aware of the limitation and consequences on student and assessor behaviour. Prioritising objectivity above context by trying to insist that examiners disregard their own experience and stick rigidly to a schema is likely to be unhelpful.

### Limitations

There are several limitations to this study. Despite efforts to recruit examiners with a broad range of level of experience, the majority of participants were well experienced examiners.

The study was not a real-life scenario and assessors watched each video twice, which may have affected assessor behaviour, it is well known that context has an important influence on rating. However, this neutral setting without extraneous context may also have allowed examiners the ability to be honest about how they form judgements as there were no repercussions. Watching each video twice allowed time for reflection. Many commented that they had learned something. It is also likely, due to social distancing requirements associated with COVID-19, that this type of assessment with raters making their assessments remotely, and without extraneous context, may become more common.

### Study implications

This study further reinforced the findings that rater idiosyncrasies can lead differing priorities that come into consideration during rating. In particular, raters consider OSCEs to be fundamentally flawed and seek glimpses of authenticity on which to base their judgements.

The finding that many experienced examiners are comfortable deviating from instructions provides useful insight to medical educators and anyone involved in assessments using raters.

## Conclusion

This study gave an insight into how raters approach OSCEs, how they deal with perceived shortcomings of OSCEs, examiners (perhaps the more experienced group) may deliberately deviate from an organisations instructions because of perceived shortcomings in the system. No method of assessment is perfect, and it is important to be aware of the broader consequences of the limitations and shortcomings of assessment methods on student performance and examiner perception. Further study of assessor and student perception of OSCE performance would be helpful.

## Data Availability

The datasets used during the current study is available from the corresponding author on reasonable request.
